# Impacts of Agricultural Management and Climate Change on Future Soil Organic Carbon Dynamics in North China Plain

**DOI:** 10.1371/journal.pone.0094827

**Published:** 2014-04-10

**Authors:** Guocheng Wang, Tingting Li, Wen Zhang, Yongqiang Yu

**Affiliations:** State Key Laboratory of Atmospheric Boundary Layer Physics and Atmospheric Chemistry, Institute of Atmospheric Physics, Chinese Academy of Sciences, Beijing, China; DOE Pacific Northwest National Laboratory, United States of America

## Abstract

Dynamics of cropland soil organic carbon (SOC) in response to different management practices and environmental conditions across North China Plain (NCP) were studied using a modeling approach. We identified the key variables driving SOC changes at a high spatial resolution (10 km×10 km) and long time scale (90 years). The model used future climatic data from the FGOALS model based on four future greenhouse gas (GHG) concentration scenarios. Agricultural practices included different rates of nitrogen (N) fertilization, manure application, and stubble retention. We found that SOC change was significantly influenced by the management practices of stubble retention (linearly positive), manure application (linearly positive) and nitrogen fertilization (nonlinearly positive) – and the edaphic variable of initial SOC content (linearly negative). Temperature had weakly positive effects, while precipitation had negligible impacts on SOC dynamics under current irrigation management. The effects of increased N fertilization on SOC changes were most significant between the rates of 0 and 300 kg ha^−1^ yr^−1^. With a moderate rate of manure application (i.e., 2000 kg ha^−1^ yr^−1^), stubble retention (i.e., 50%), and an optimal rate of nitrogen fertilization (i.e., 300 kg ha^−1^ yr^−1^), more than 60% of the study area showed an increase in SOC, and the average SOC density across NCP was relatively steady during the study period. If the rates of manure application and stubble retention doubled (i.e., manure application rate of 4000 kg ha^−1^ yr^−1^ and stubble retention rate of 100%), soils across more than 90% of the study area would act as a net C sink, and the average SOC density kept increasing from 40 Mg ha^−1^ during 2010s to the current worldwide average of ∼55 Mg ha^−1^ during 2060s. The results can help target agricultural management practices for effectively mitigating climate change through soil C sequestration.

## Introduction

Cultivation generally leads to dramatic changes in soil organic carbon (SOC) by influencing the processes involved with soil C production and decomposition [1], [2]. Changes in agricultural SOC are characterized by dynamic exchange processes which is strongly linked to environmental conditions such as air temperature, precipitation, soil pH and texture; and agronomic management such as crop rotation, stubble retention, tillage regimes, and application of chemical fertilizers and animal manure [3], [4]. The complex interactions between management practices and environmental conditions, as well as the lack of spatiotemporal continuity in SOC monitoring data over meaningfully large areas, hamper our ability to accurately predict the regional cropland SOC change and identify the factors that control the SOC dynamics.

A modeling approach has advantages in estimating spatiotemporal changes in SOC under various management practices and environmental conditions [5]. A number of process-based carbon models such as DNDC [6], RothC [7], and CENTURY [8] have been used to simulate SOC dynamics in agricultural systems at both national and continental scales [9]–[11]. Recently, Huang et al. [12] developed a biogeophysical model, Agro-C, which has been validated and further used to assess the long-term agricultural SOC changes on a national scale in China [5] and Australia [2].

**Figure 1:**
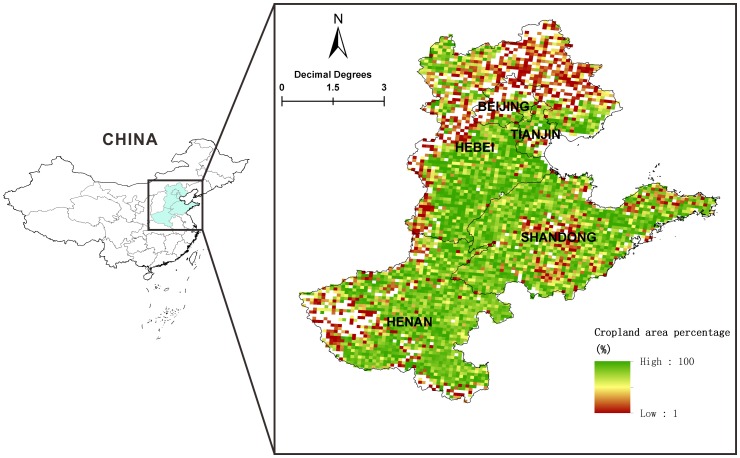
Spatial distribution of croplands in the North China Plain.

The North China Plain (NCP) is one of the most important agricultural production areas in China, covering a total cropland area of ∼18 Mha, with a typical continuous winter wheat-summer maize cropping system (Figure 1). During the past several decades, significant changes in both climate and agricultural management practices have taken place in NCP. For example, Ding et al. [13] reported that both temperature and precipitation had shown an increasing trend across the northern part of China during the past 50 years. Improved management practices such as crop residue retention and application of mineral fertilizers and/or organic manure have been promoted as strategies to both increase crop productivity and optimize soil fertility [14]. Although an overall net increase in SOC across the croplands of NCP during the past 30 years has been reported [5], there remains large uncertainties in future cropland SOC dynamics due to the unclear future changes in both climate and management practices. For instance, it is generally recognized that the current global warming will increase the rates of organic matter decomposition, thereby accelerating climate change through C cycle feedbacks [15]. However, in areas with relatively low mean annual temperature and without water and nutrient deficiencies (e.g., the North China Plain, typically with irrigation and N fertilization to support crop production), increasing air temperature could potentially increase crop productivity and result in more input C into soils, thereby offsetting the loss in SOC through soil respiration. More recently, Yu et al. [16] simulated the Chinese cropland SOC changes during the next 40 years, by simply designing three levels of carbon input based on the historic changing trend in annual amounts of crop residues and manure. However, the quantity of input C is strongly linked to management such as stubble retention and N fertilization, which needs to be considered during a comprehensive assessment of long-term SOC dynamics, and determined to provide strategies in effectively managing the farming systems. Moreover, since it takes several decades or even a century to reach a new equilibrium of SOC following a change in management practice [17], [18], the role of soils to sequester C (source or sink) as affected by environmental variables and management practices needs to be identified on a long time scale, e.g., around 100 years.

In this study, we quantified the influence of both climatic variables and management practices on SOC across croplands of North China Plain. The SOC was simulated under 900 combinations of climate, N fertilization, stubble retention, and manure application scenarios at a daily time step, from 2011 to 2100, and at a high spatial resolution, using the pre-calibrated and validated Agro-C model. The impacts of management practices and climatic variables on SOC dynamics were then statistically analyzed. This would not only provide a better understanding of the factors and processes regulating C cycling and balance in the agro-ecosystems, but would also provide insight into the effectiveness of methods for enhancing soil quality and mitigating climate change.

## Materials and Methods

### 1.1 Agro-C

Agro-C [12] is a biogeophysical model, and it consists of two submodels, Crop-C and Soil-C. The Crop-C submodel simulates processes involved with crop photosynthesis, autotrophic respiration, and net primary production (NPP); and the Soil-C submodel simulates soil heterotrophic respiration via the decomposition of both input C (e.g., crop residues, roots and manure) and SOC. Changes in SOC are then determined by balancing the loss of SOC with the gain of input C. The input C is split into two components of labile-C and resistant-C, and the SOC pool is divided into two sub-pools named light-C and heavy-C. The light-C sub-pool is hypothesized to be more biologically reactive, while the heavy-C sub-pool is much more resistant to decomposition. Decomposition of each component and sub-pool is described by first-order kinetics at predefined potential rates (d^−1^) of 2.6×10^−2^, 8.4×10^−4^, 2.5×10^−4^ and 1.8×10^−5^ for labile-C, resistant-C, light-C and heavy-C, respectively. The actual decomposition rate of each component and sub-pool is further modified by soil parameters such as temperature, moisture, texture and pH [12]. The C flow between different pools was determined based on the following assumptions: (i) the decomposition of both the labile-C and the resistant-C converts a fraction of the C into the light-C pool, (ii) a fraction of the decomposed light-C is transferred into the heavy-C pool, and (iii) the decomposition of the heavy-C pool only produces CO_2_. More detailed description of the Agro-C model was shown in Yu et al. [5] and Huang et al. [12].

In our previous studies [5], [12], Agro-C has already been parameterized using observed data of several field measurements, and validated against independent datasets across a vast area representing different cropping systems, soil and climate conditions in China. The results indicate that the calibrated Agro-C model can reasonably capture observed aboveground biomass and changes in SOC under various agricultural management practices at different sites across china [5], [12]. In the present study, we used the previously calibrated Agro-C model to simulate regional SOC dynamics of agro-ecosystems in North China Plain (NCP).

### 1.2 Spatial data

The changes in SOC under different climatic and management scenarios were simulated with a daily step from 2011 to 2100. Up-scaling of the Agro-C model was accomplished by first rasterising the model inputs (e.g., climate data, initial soil properties and agricultural management information) with 10 km×10 km gridded datasets across the North China Plain and then running the model grid by grid across the study area.

Input soil properties in Agro-C include the concentrations of organic carbon and total nitrogen, bulk density, clay and sand fraction, and pH in the topsoil to 30 cm depth. These soil properties, with a 10 km×10 km spatial resolution, were firstly obtained from China Soil Scientific Database [19], representing more than 7000 soil profile measurements across China made in the late 1970s/early 1980s. Using Agro-C, we then simulated these soil properties grid by grid on a daily step from 1981 to 2010, based on the historical climate data and recorded agricultural management practices [5]. The model outputs of soil properties, including the amount of C in different pools, in the end of 2010 were further compiled as the initial model input of soil properties of the present study.

Gridded daily climate data such as maximum and minimum temperature, precipitation, and relative humidity from 2010 to 2100 were derived from the projections of the climate model FGOALS (Document S1). Due to the coarser spatial resolution (2.8° longitude×2.8° latitude) of outputs from the FGOALS model, the projections for the future GHG concentration scenarios [i.e., a set of four representative concentration pathways (RCPs) named RCP2.6, RCP4.5, RCP6.0 and RCP8.5 respectively; Figure S1 and Figure S2] of the FGOALS were therefore statistically downscaled [20], [21] to a resolution of 10 km×10 km (Document S1). Additionally, as a driving variable in Agro-C, atmospheric carbon dioxide (CO_2_) concentration affects the process of crop photosynthesis [12], which under each future climate scenario was obtained from Clarke et al. [22] and used as model inputs in this study.

The spatial distribution of croplands was computed based on the National Land Cover Data Sets (NLCD) of China that was developed from Landsat TM digital images around 2000 [23]. Due to a lack of yearly cropland data with sufficient spatial distribution, we assumed that the area of croplands in North China Plain did not significantly change over the study period.

### 1.3 Model initialization

In Agro-C model, the half-life residence time for labile-C, resistant-C, light-C and heavy-C were 0.1 y, 2.3 y, 7.6 y, and 105.4 y [5], respectively. To obtain initial fractions for the different C pools in 1981, a ‘spin-up’ procedure was performed by first setting the initial fractions of light-C and heavy-C as 0.25 and 0.75 [5], respectively, and then running the model until a steady state of SOC pool was achieved. In this, an equilibrium run was not stopped until the difference of SOC pools between two successive years was less than 0.1% of the pools themselves. The length of spin-up procedure differed across different places, and generally lasted about 100 years. The average time duration of the spin-up seemed relatively short but is reasonable. Most of Chinese croplands, particularly in North China Plain, have generally experienced a very long history of cultivation. When the spin-up procedure was performed, the average amount of crop stubble retention and manure amendment over the period between 1980 and 1989 was used as organic C input, due to the unavailability of organic C input before 1980. The average of 1960–1990 climate were used as driving data, because of the unavailability of climate data before 1960. The other driving datasets such as nitrogen fertilizer and irrigation were set to 1980–1989 average values. We then calculated the initial values of different C pools based on the total SOC information obtained from the above mentioned soil database and the spin-upped fractions of different sub-C pools. The dynamics of different C pools in each grid from 1981 to 2010 on a daily time step were then modeled based on the historical climate data and recorded agricultural management practices [5]. The simulated values of above-mentioned four C pools in the end of 2010 were further compiled as the model inputs of the present study.

### 1.4 Agricultural management scenarios

Nitrogen (N) fertilization, application of manure, and crop stubble retention through tillage are the major management practices that affect SOC dynamics in croplands of North China Plain (NCP). A database containing the county-level yearly cultivation acreage and total amount of synthetic N application (Chinese Academy of Agricultural Sciences [24]), shows that N fertilization rates in 2010 across croplands of NCP varied from 0 to 800 kg ha^−1^ (Figure S3A). The environmental pressures that are related to excessive mineral N fertilization in Chinese croplands decrease the possibility of increasing its use in the future [25]. Another province-level database [26] shows that farm manure application rates during the past 30 years varied from 1000 to 4000 kg ha^−1^ across the studied area (Figure S3B). In the croplands of NCP, the average stubble retention rate over the period of 2000–2010 varied from ∼40% to ∼60% across space [27]. In general, an increasing fraction of crop residues were being left in the fields instead of being taken away for household fuel and animal feed in China over the past several decades [25], which was mainly attributed to the financial motivations of the government policies. To quantify the effects of both climate change and agricultural management practices on SOC, we simulated SOC changes within the winter wheat-summer maize systems under 225 combinations of N fertilization, manure application and stubble retention rates, under each of the above-mentioned four climate scenarios.

In this study, management practices included 9 nitrogen application rates (0–800 kg N ha^−1^ yr^−1^ in 100 kg N ha^−1^ yr^−1^ increments, i.e., N:0, N:100, N:200, N:300, N:400, N:500, N:600, N:700, and N:800). Apart from N:0, we specified that 55% of the N fertilizers were applied during summer-maize growing seasons, with another 45% applied during winter-wheat growing seasons. For each growing season, 40% of the N fertilizers were applied at crop emergence, another 40% applied at tillering, and the rest applied at heading for wheat and silking for maize. We also specified five manure application rates (0–4000 kg ha^−1^ yr^−1^ in 1000 kg ha^−1^ yr^−1^ increments, i.e., M:0, M:1000, M:2000, M:3000, and M:4000), and five stubble retention rates (0–100% in 25% increments, i.e., R:0, R:25, R:50, R:75, and R:100). Apart from M:0, 72% of the manure was applied before sowing of summer maize, with the rest applied before sowing of winter wheat. Stubble retention rates denoted the percentage of aboveground straw and leaf biomass incorporated into the soil by tillage after harvesting, with the rest directly removed from the system. Combinations of management practices are abbreviated in this study as M:x, N:x, R:x. The M:0, N:0, R:0 denotes the management practice with no N fertilization, no manure application, and no stubble retention.

Because of a lack of detailed crop cultivar and phenology data on the regional scale, we simply assumed that the same wheat and maize cultivars were used throughout the studied area, and crop parameters were set as default values of the pre-calibrated Agro-C model. Sowing dates of maize and wheat were based on local crop calendars. From the south to north in NCP, the sowing date ranged from 6 June to 15 June for maize, and ranged from 5 October to 11 November for wheat [28], [29]. Crop growth stops at physiological maturity as determined by the accumulated temperature. In addition, according to the local agronomic management, we assumed that the soil moisture would arrive at field capacity using irrigation module when the modeled soil moisture in the 30 cm depth drops to 70% of the field capacity during the growing seasons [5].

In total, we ran 4,086,000 (225 management scenarios ×4 climate scenarios ×4540 grids) Agro-C simulations. Each simulation quantified SOC content in the top 30 cm of soil from 2011 to 2100 on a daily step.

### 1.5 Identifying controls on SOC dynamics

In the present study, we assessed the impacts of management practices, climatic and soil variables on SOC change using Spearman's rank correlation coefficient (*rho*). Selected climatic variables included mean annual temperature (hereafter simply denoted as *temperature*) and mean annual precipitation (hereafter simply denoted as *precipitation*) since these two variables have been reported uncorrelated and significantly represent the spatial variation in a range of climate patterns [30]. For correlation analysis, the long-term daily climate variables were summarized to mean annual values under each climate scenario for each grid. Selected soil parameters included the main model edaphic inputs, e.g., pH, initial SOC, clay and total nitrogen content. Change in SOC of the top 30 cm (ΔSOC) is calculated as the difference in SOC between 2100 and 2011. Spearman's rank correlation coefficient was then calculated between ΔSOC and the management practices and environmental variables across the full set of Agro-C simulations. The sign of *rho*, positive or negative, indicates the direction of association between the independent and dependent variables. The absolute magnitude of *rho*, between 0 and 1, suggests the strength of correlation between the two variables.

We also investigated the effects of the initial SOC content and each of the three management practices on △SOC by producing boxplots characterizing the influence of each variable on △SOC including the variance calculated across agricultural management practices, grids, and the four climate scenarios. All analyses were performed using statistical and graphical software R 3.0.1 [31].

## Results

### 2.1 Correlations between environmental variables, management practices and △SOC

**Figure 2:**
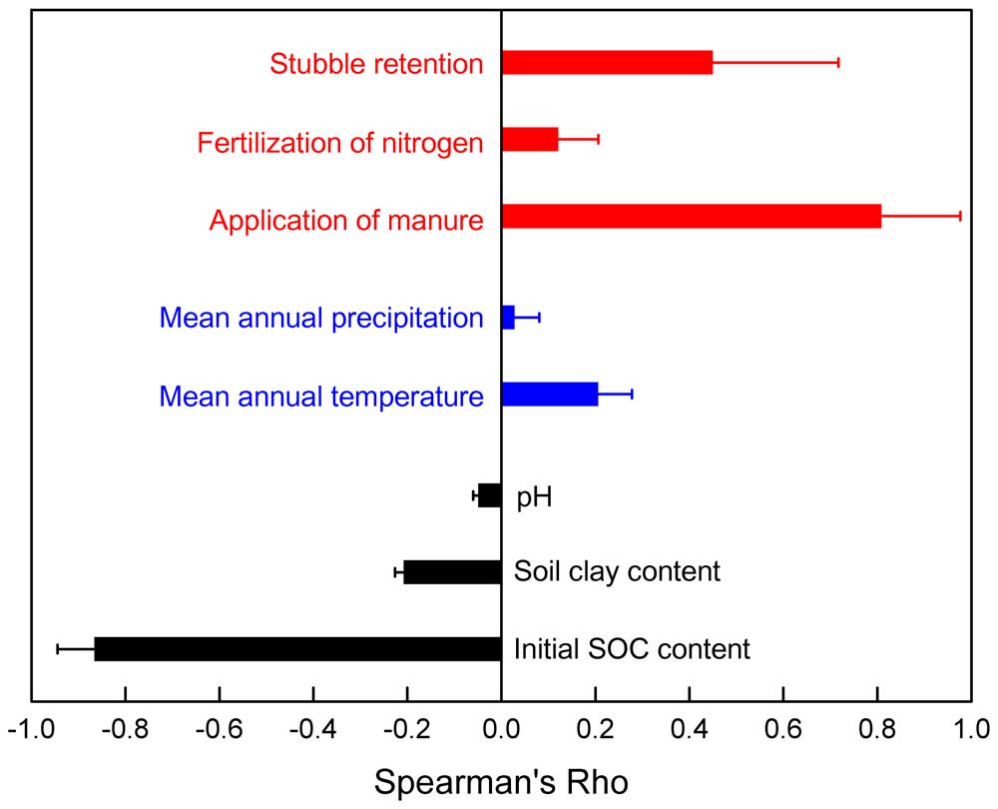
Spearman's rank correlation coefficients between ΔSOC (2011–2100, Mg C ha^−1^) and climate (blue), soil (black), and management (red) variables. All the tests were significant (*P*<0.001).

Initial SOC was strongly but negatively correlated (median *rho*  = −0.87) with △SOC (Figure 2). Initial soil clay content showed a weak negative correlation (median *rho*  = −0.21) with △SOC (Figure 2). The climatic variables, e.g., temperature and precipitation, displayed a weak positive (median *rho*  = 0.21) and negligible correlation with △SOC, respectively (Figure 2). Agricultural management such as stubble retention (median *rho*  = 0.45) and manure application (median *rho*  = 0.81) both showed a strong and positive correlation, whereas N fertilization showed a negligible correlation with △SOC (Figure 2).

**Figure 3:**
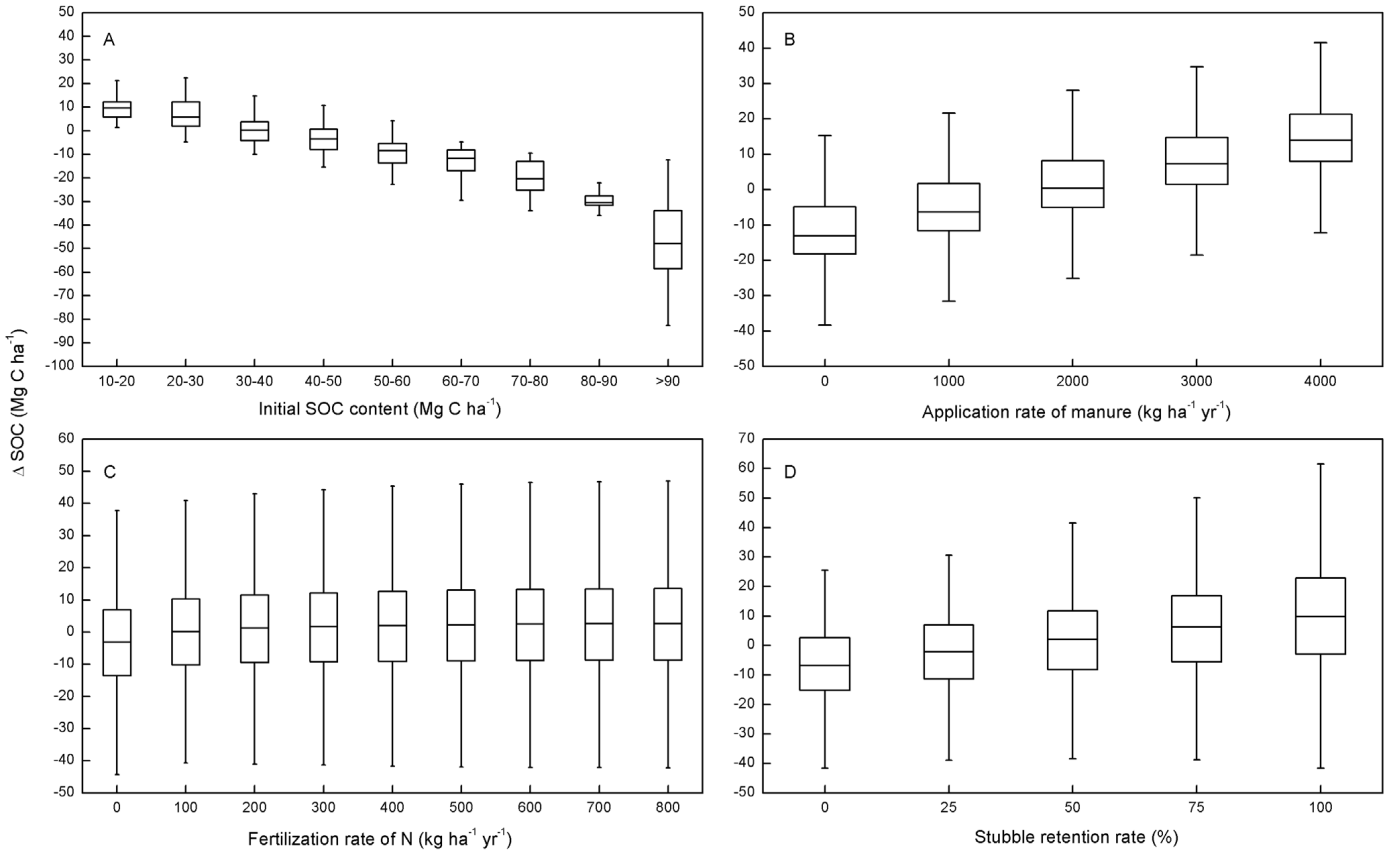
Response of ΔSOC (2011–2100, Mg C ha^−1^) to initial SOC content (A), manure application (B), N fertilization (C), and stubble retention (D). Boxplots show the median and interquartile range, with whiskers extending to the most extreme data point within 1.5×(75–25%) data range.

**Figure 4:**
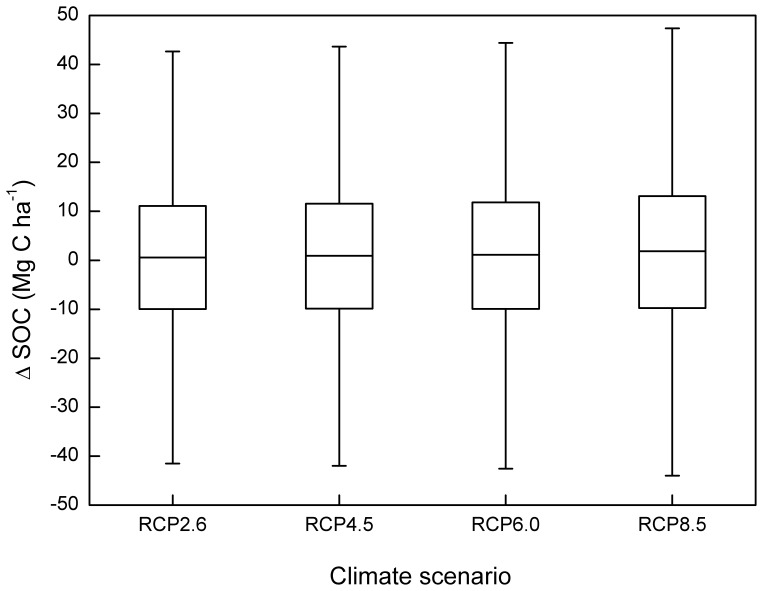
Response of ΔSOC (2011–2100, Mg C ha^−1^) to different climate scenarios. Boxplots show the median and interquartile range, with whiskers extending to the most extreme data point within 1.5×(75–25%) data range.

Figure 3 presented the impacts of initial SOC content, manure application, N fertilization and stubble retention, respectively, on △SOC. On average, initial SOC content seemed to linearly and negatively correlate with △SOC (Figure 3A), whereas manure application and stubble retention had a relatively linear positive effect (Figure 3B and D) on △SOC. N fertilization increased △SOC most significantly (*P*<0.05) at rates between 0 and 300 kg ha^−1^ yr^−1^, with a limited influence at higher fertilization rates. Across the sets involved with different climate scenarios, the median △SOC increased slightly, although not significantly (*P*>0.05), from RCP2.6 to RCP8.5 (Figure 4).

### 2.2 Spatiotemporal effects of agricultural practices on SOC

**Figure 5:**
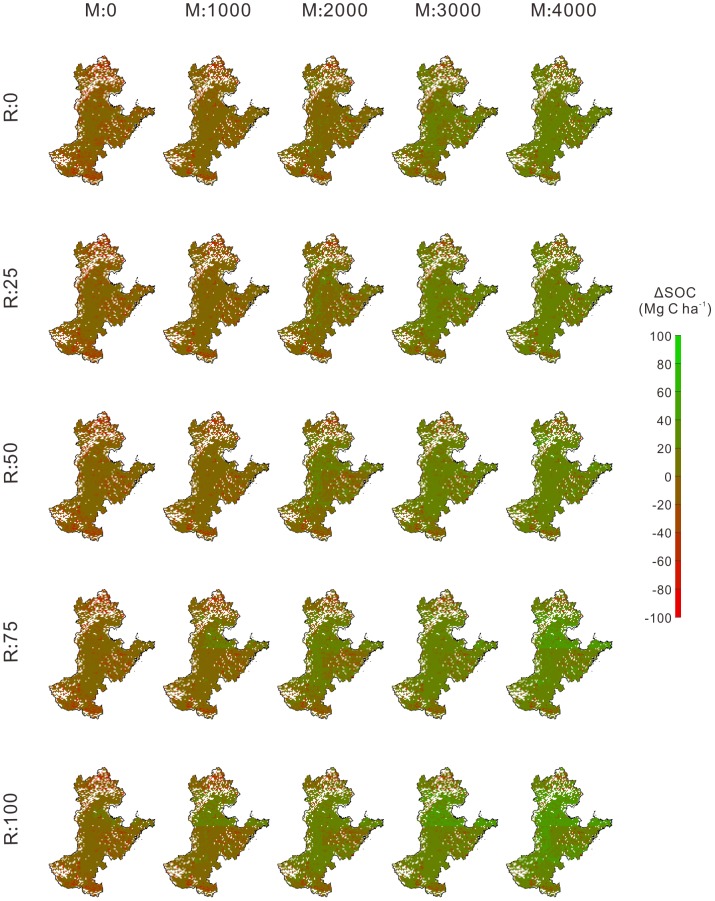
Spatial pattern of the effects of manure application and stubble retention on ΔSOC (2011–2100, Mg C ha^−1^) under N:0. For a certain combination of management practices, ΔSOC in each grid represents the mean value of the results under four climate scenarios.

**Figure 6:**
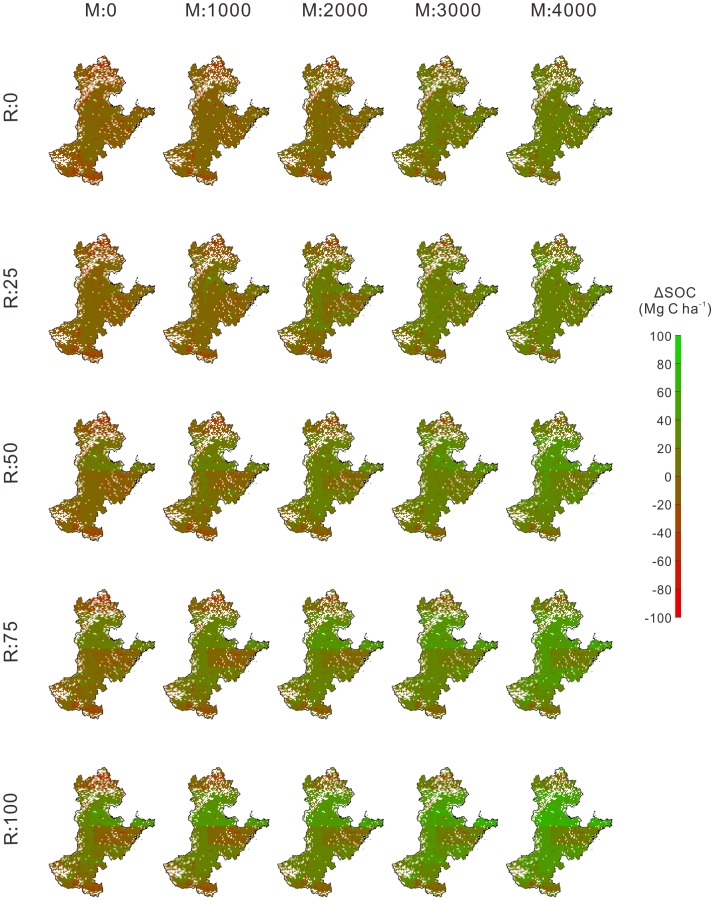
Spatial pattern of the effects of manure application and stubble retention on ΔSOC (2011–2100, Mg C ha^−1^) under N:300. For a certain combination of management practices, ΔSOC in each grid represents the mean value of the results under four climate scenarios.

On average of the four climate scenarios, the response of △SOC to N fertilization, manure application and stubble retention varied over the study area, and cropland soils in Hebei and northern part of Shandong Province would accumulate more carbon than the rest of the study area if agricultural practices were optimized (Figure 5 and 6). Increasing the manure application rate led to soil across more areas turning into a net C sink, regardless of the other two management practices (Figure 5 and 6). N fertilization could not significantly halt the declining SOC trend unless more crop residues were incorporated into soils. For example, when stubble retention rate increased from 0 to 100% under M:2000, SOC increased in 11.4%, 27.7%, 40.7%, 53.9%, and 63.9% of the study area under N:0 (Figure 5), whereas increased in 13.5%, 44.5%, 62.5%, 71.3%, and 76.6% of the study area under N:300 (Figure 6). If the amount of manure application rate reached 4000 kg ha^−1^ yr^−1^ and all stubble incorporated into soils (i.e., M:4000 and R:100), SOC increased in more than 90% of the study area under both N:0 and N:300 (Figure 5 and 6).

**Figure 7:**
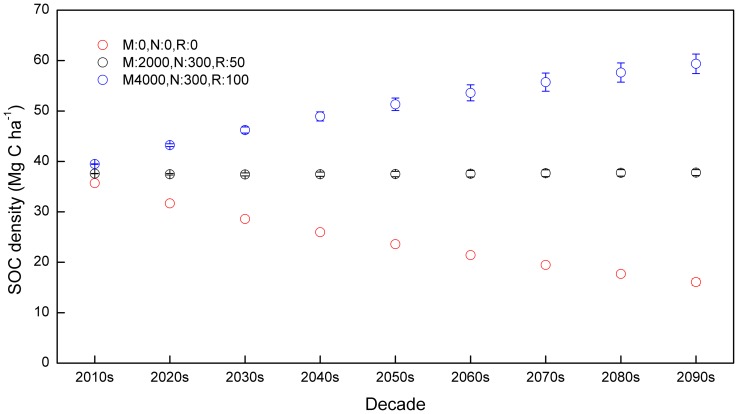
Temporal change of SOC (Mg C ha^−1^) under three combinations of manure application, nitrogen fertilization and stubble retention. Open circles represent mean values of SOC under four climate scenarios, with standard deviations represented by error bars on the circles.

Under M:0, N:0, R:0, the average SOC content across croplands of NCP kept decreasing from 35.7 Mg ha^−1^ during the 2010s to 16.1 Mg ha^−1^ during the 2090s, on average of the four climate scenarios (Figure 7). However, adopting a moderate management practice (e.g., M:2000, N:100, R:50) could maintain the SOC level during the next 90 years (Figure 7). Under M:4000, N:300, R:100, the average SOC density kept increasing from 39.5 Mg ha^−1^ during the 2010s to 54.6 Mg ha^−1^ during the 2060s, with a relatively lower increasing rate afterward (Figure 7).

## Discussion

### 3.1 Interpretation and implication of the results

Initial SOC content was identified as strongly and negatively influencing SOC change (Figure 2). This is because, in model initialization, organic C input were taken to be the average of crop stubble and manure from 1980 to 1989. However, in the following model simulations with various kinds of management scenarios, great land use changes took place and the equilibrium of soil C pools got changed. In this, under otherwise similar environmental and managed conditions, soils with higher initial SOC content would experience greater SOC loss, and vice versa [32]. This negative correlation between △SOC and initial SOC content has also been documented in other studies [33]–[35]. Although higher soil clay content has been reported to benefit the stabilization of soil organic matter (SOM) in many studies [36]–[38], we identified a weak negative correlation between △SOC and soil clay content in the present study (Figure 2). This is because, other than the benefits of SOM stabilization, higher soil clay content could also reduce the amount of C inputs to soil [12], thereby showing a negative impact on total SOC variations in the present study.

In Agro-C, high soil temperature and soil water content increase the SOC decomposition rate which underpins the negative relationship of SOC accumulation with both temperature and rainfall [12]. However, in NCP, we found that the mean annual temperature and precipitation were not strongly correlated with △SOC (Figure 2), and higher temperature seemed to benefit SOC accumulation (Figure 4 and Figure S1). This is because the influences of temperature and precipitation on SOC dynamics depend on other factors such as soil and management practices [39]. For example, artificial irrigation, which was adopted across all sets of scenarios in this study, would certainly overshade the possible impacts of precipitation on SOC change. And the elevated atmospheric CO_2_ concentration accompanied with higher temperature increased the crop productivity, thereby compensating the elevated amount of decomposed SOC induced by climate warming [40]. Furthermore, Zhang and Huang [41] reported a positive correlation between air temperature and the production of cereal crops in China during the past 30 years. This indicated that climate warming could potentially cause a higher amount of C input from crop residues to soils, with which our findings was consistent.

Manure application and stubble retention directly add C into soil, and were identified as the most two predominant agricultural management practices driving SOC (Figure 2 and 3). Although it has been reported that the manure application levels has been decreasing during the past 50 years, and the changing trends might continue in the future across Chinese croplands [25]. The increasing amount of incorporated crop residues, caused by both enhanced stubble retention rate [27] and elevated amount of crop NPP [42], could still potentially override the weakening manure application rates and increase SOC.

**Figure 8:**
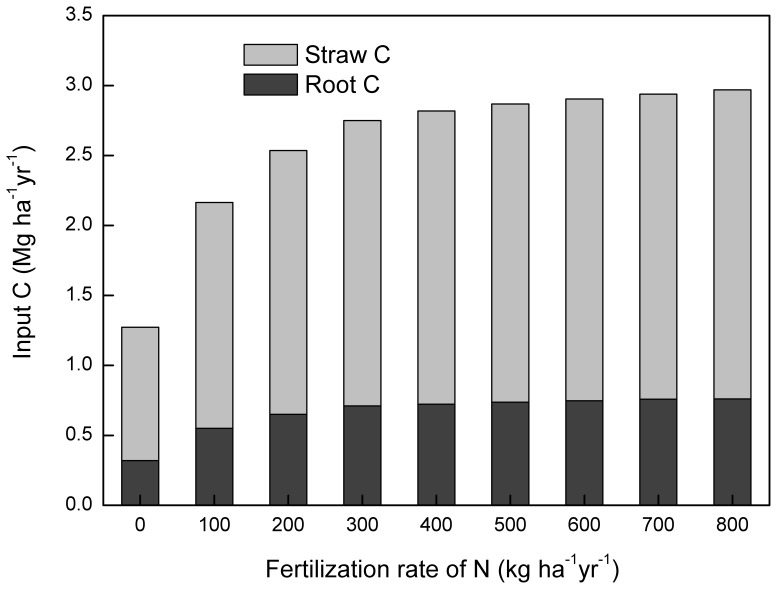
Response of mean annual C inputs from crop residues and roots (Mg C ha^−1^) to different N fertilization rates.

N fertilization enhances crop residue and root biomass production (Figure 8), thus increases the amount of C incorporated into soil. However, the impact of N fertilization depends on whether nutrient is limiting. In this study, we found that N fertilization showed a negligible correlation with changes in SOC (Figure 2). This is mainly because changes in SOC is dominated by manure application and stubble retention in most of the scenarios. However, under scenarios with high stubble retention rates and low manure application rates, effects of N fertilization could be more obvious (Figure 5 and 6). Additionally, our results also showed that although N fertilization had a positive effect on SOC accumulation, the effect did not further increase if N fertilization rate exceeds 300 kg ha^−1^ yr^−1^ (Figure 3C). This is because with a N fertilization rate higher than 300 kg ha^−1^ yr^−1^, nutrient was not a limiting factor of crop growth, and enhancing N inputs could not significantly elevate the amount of soil C inputs from crop residues and roots (Figure 8). Additionally, excessive use of N fertilizer is currently very common in NCP [43], which has aroused concerns about its negative impacts on ecosystems and environments [44]. We suggest that a N fertilization rate of about 300 kg ha^−1^ yr^−1^ should be adopted to both maintain SOC accumulation and reduce the environmental risks caused by excessive use of N fertilizers. As the benefit of N fertilization on SOC sequestration could be offset by N_2_O emissions from the soil and greenhouse gas (GHG) emissions from fossil fuel combustion during the processes involving fertilizer production and transport [45], there remains a need to further investigate the proper rates and timing of N fertilization under different climate and soil conditions.

With a high (M:4000, N:300, R:100) C input management, the temporal change in SOC generally experienced a fast to low increase, with the most gain of soil C happened in the first 50 years of simulations (Figure 7). This is consistent with a number of other studies [1], [46], which suggest that soil C would reach a new steady state after 20–50 years of cultivation if management practices were significantly changed. Moreover, our results revealed that under M:4000, N:300, R:100, the average SOC density of NCP croplands could achieve 55 Mg ha^−1^ during 2060s, which is similar to the current worldwide average agricultural SOC density [47]. However, the average SOC density would keep decreasing under M:0, N:0, R:0, and the “new” steady state of SOC was not attained even after 90 years of simulation (Figure 7). This could be attributed to the limited C input to balance the SOC loss by decomposition. With a much lower decomposition rate, the proportion and absolute amount of heavy-C would have significant impact on long-term SOC balance.

### 3.2 Uncertainties and limitations

Several uncertainties and limitations should be noticed in interpreting the simulation results in this study. First, several management scenarios simulated in this study represent the most extreme conditions and may not happen in the real world. For example, zero inputs of N and C to soil, because it is improbable to extract all roots and residues from the fields, or prevent atmospheric N deposition. Second, the possible improvement in future crop varieties, which could potentially affect the quality and quantity of soil C inputs, was not considered in this study. For example, if the crop variety improvement increased the production of either crop residues or roots, this could lead to an underestimation of our results in the future SOC accumulation. Third, the effects of conservation tillage on SOC were not simulated in this study, due to a lack of the detailed information on annual conservation tillage practice. However, it could still be hypothesized that our results could represent most parts of the studied areas, because the average fraction of the area with conservation tillage practices in Chinese croplands is only about 9% in 2009 [5]. Moreover, a meta-analysis of global data on the effects of tillage on SOC change [48] indicates that conversion from conventional tillage to conservation tillage only redistributed more C into the top layer and less C into the deeper layer, and the total SOC stock in the upper 40 cm soil profile was not significantly changed. Fourth, the rainfed condition was not simulated because artificial irrigation has been traditionally adopted to support crop production in NCP. However, the current irrigation-supported agricultural production might be affected by future resource and environment related pressures [49]. In this, other environmental factors such as precipitation and temperature may play a more important role in regulating both crop productivity and SOC dynamics [50]. Last but not least, certain issues related to state-of-the-art model limitations should be mentioned. For instance, in a first-order decay model (e.g., Agro-C), there is a general linear relation between C input and SOC variation, which usually contradicts the fact that increase of C inputs by crop residues may have variable effects on SOC change in the real world [51]. Future efforts are needed from model developers to implement currently uncertain mechanisms, such as SOC saturation, priming, and different sensitivity of SOC pools to temperature, which will enable a better understanding the influence of environment on agricultural SOC dynamics.

## Supporting Information

Figure S1 **Climate change in North China Plain (NCP) from 2011 to 2100 under the RCP2.6 (A), RCP4.5 (B), RCP6.0 (C) and RCP8.5 (D) scenarios.** The solid circles represent the annual temperature, and the open triangles represent annual precipitation.
(TIF)

Figure S2 **Spatial distribution of mean annual temperature and precipitation increments in North China Plain (NCP) between 2011 and 2100 under four climate scenarios.**
(TIF)

Figure S3 **The range and density of N fertilization (A) and manure application (B) rates in North China Plain (NCP).** The N fertilization data represent the year of 2010 on a county scale, and the manure application data represent that from 1981 to 2010 on a provincial scale. The datasets were derived from online database and published literatures. *n* shows the sample size.
(TIF)

Document S1 **Description of FGOALS and the RCP scenarios for AR5.**
(DOC)
