# Supplementary figures and images for: Impacts of Agricultural Management and Climate Change on Future Soil Organic Carbon Dynamics in North China Plain
Source: PLoS One. 2014 Apr 10;9(4):e94827. doi: 10.1371/journal.pone.0094827 (PMC3983264; doi:10.1371/journal.pone.0094827)

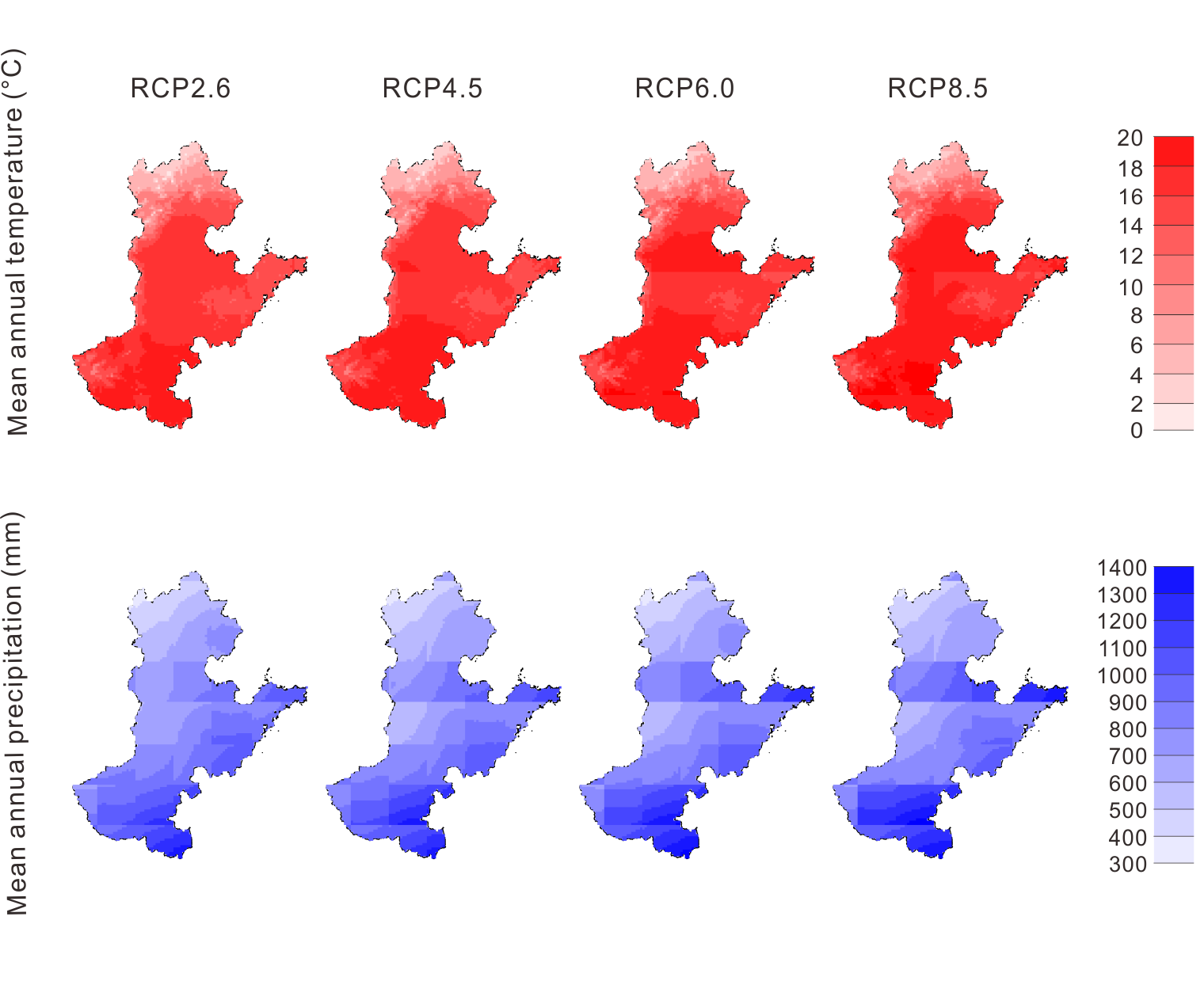

Supplement: Figure S2 — Spatial distribution of mean annual temperature and precipitation increments in North China Plain (NCP) between 2011 and 2100 under four climate scenarios. (TIF) [file pone.0094827.s002.tif]

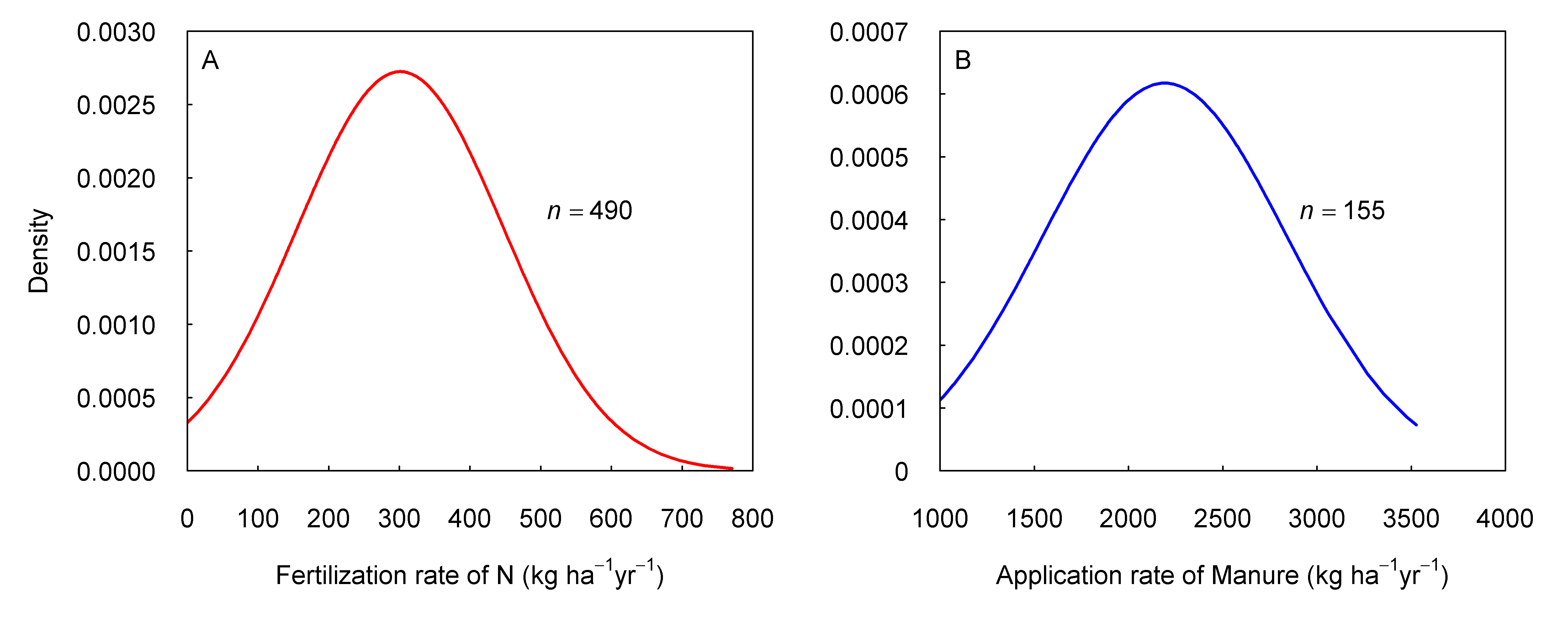

Supplement: Figure S3 — The range and density of N fertilization (A) and manure application (B) rates in North China Plain (NCP). The N fertilization data represent the year of 2010 on a county scale, and the manure application data represent that from 1981 to 2010 on a provincial scale. The datasets were derived from online database and published literatures. n shows the sample size. (TIF) [file pone.0094827.s003.tif]
