# Supplementary material for: Impacts of Agricultural Management and Climate Change on Future Soil Organic Carbon Dynamics in North China Plain
Source: PLoS One. 2014 Apr 10;9(4):e94827. doi: 10.1371/journal.pone.0094827 (PMC3983264; doi:10.1371/journal.pone.0094827)
Supplement: Document S1 — Description of FGOALS and the RCP scenarios for AR5. (DOC) [file pone.0094827.s004.doc]

**S1. Description of FGOALS and the RCP scenarios for AR5**

The Flexible Global Ocean-Atmosphere-Land System model (FGOALS) is a GCM (General Circulation Model), which was developed by the Institute of Atmospheric Physics at the Chinese Academy of Sciences (IAP, CAS), and has contributed to the 4^th^ assessment report (AR4) of the IPCC . Its latest version has been ﬁnalized for participation in 5^th^ phase of the Coupled Model Intercomparison Project (CMIP5), and the model results have been submitted to CMIP5 for inclusion in the 5^th^ Assessment Report (AR5) of the IPCC . Because the projections of climate change depend heavily upon future human activity, climate models are run against scenarios. In total, a set of four representative concentration pathways (RCPs) were grouped from a number of scenarios in the existing related literatures . These four RCPs include one mitigation scenario leading to a very low forcing level (RCP2.6), two stabilization scenarios (RCP4.5 and RCP6.0), and one scenario with very high greenhouse gas emissions (RCP8.5). These scenarios include time paths for emissions and concentrations of the full suite of greenhouse gases (GHGs) and aerosols and chemically active gases, as well as land use/land cover, and have been reported in the IPCC expert meeting report . In this study, we used the projections of FGOALS model for each of the four scenario, which represented a balanced emphasis on all of the energy sources and an ecologically integrated world.

The outputs of the FGOALS for future climate change projections are on a daily step, including the maximum and minimum temperatures, precipitation and solar radiation, with a coarse spatial resolution of 2.8° × 2.8° for the geographical longitude and latitude. Thus, a statistical down scaling approach was adopted to downscale the climate projections. Firstly, regression equations were established to create relationships between each 10 km ×10 km grid and its enclosing 2.8° × 2.8° grid with the data from the overlapping period of 1960 to 1990. The regression equations were then applied to the FGOALS outputs to calculate the daily climatic data for the 10 km ×10 km grid from 2011 to 2100.

Figure S1 shows the changes in the annual mean air temperature and precipitation in North China Plain from 2011 to 2100, with future climatic data projected by the FGOALS. Under the RCP2.6, the radiative forcing level first reaches a value of around 3 W m^-2^ (~ 490 ppm CO_2_ eq) by mid-century and returns to 2.6 W m^-2^ (~ 425 ppm CO_2_ eq) by 2100. The RCP4.5, RCP6.0 and RCP8.5 scenarios set the radiative forcing level keeping increase to 4.5 W m^-2^ (~ 650 ppm CO_2_ eq), 6.0 W m^-2^ (~ 850 ppm CO_2_ eq) and 8.5 W m^-2^ (~ 1370 ppm CO_2_ eq), respectively, by 2100. The annual mean air temperature and precipitation under each climate scenario were presented in Figure S1. Spatially, under each climate scenario, projected temperatures all showed an increasing trend from north to south of NCP, while precipitation showed an decreasing trend from costal areas of the east to inland areas of the west (Figure S2).

**Reference**

1. Yu YQ, Yu RC, Zhang XH, Liu HL (2002) A flexible coupled ocean-atmosphere general circulation model. Advances in Atmospheric Sciences 19: 169-190.

2. Yu YQ, Zhang XH, Guo YF (2004) Global coupled ocean-atmosphere general circulation models in LASG/IAP. Advances in Atmospheric sciences 21: 444-455.

3. Li L, Lin P, Yu Y, Wang B, Zhou T, et al. (2013) The flexible global ocean-atmosphere-land system model, Grid-point Version 2: FGOALS-g2. Advances in Atmospheric Sciences 30: 543-560.

4. Van Vuuren DP, Edmonds J, Kainuma M, Riahi K, Thomson A, et al. (2011) The representative concentration pathways: an overview. Climatic Change 109: 5-31.

5. Moss RH, Babiker M, Brinkman S, Calvo E, Carter T, et al. (2008) Towards new scenarios for analysis of emissions, climate change, impacts, and response strategies. Pacific Northwest National Laboratory (PNNL), Richland, WA (US).

6. Kidson JW, Thompson CS (1998) A comparison of statistical and model-based downscaling techniques for estimating local climate variations. Journal of Climate 11: 735-753.
